# Association between risk factors and migraine in Pakistani females

**DOI:** 10.1186/s12905-023-02810-5

**Published:** 2023-12-02

**Authors:** Nida Razzak, Hina Khan, Huma Tariq, Muhammad Aslam

**Affiliations:** 1 Department of Statistics, GC University Lahore, Lahore, Pakistan; 2 Additional Controller of Examination, GC University Lahore, Lahore, Pakistan; 3 Department of Statistics, Faculty of Science, King Abdulaziz University, Jeddah, Saudi Arabia

**Keywords:** Migraine disease, Risk factors, Chi-square, Logistic regression, Association

## Abstract

**Background:**

Migraine is a typical cripple issue of the brain identified with cerebral pain which is an indication of numerous health conditions. About 18% of women (27 million) and 6% of men (10 million) are afflicted by migraine in the United States. Based on a case-control study, to explore the different risk factors, causing migraine in females and examine the association between risk factors and migraine.

**Methods:**

A sample of 1055 individuals were selected in different areas of Lahore from September 2019 to March 2020. The information was obtained by using the direct interview method and questionnaire method. Descriptive analysis, bivariate analysis and binary logistic regression analysis were carried out in data analysis.

**Results:**

Among 1055 individuals 740 cases and 315 controls were included. In a binary logistic regression model, physical activities, stress, summer season, menstruation and morning were the risk factors that cause migraine and these were found to be positively significant with the odds ratios and 95% confidence interval of odds ratios (1.399; 1.122–1.746), (1.510; 1.187–1.922), (1.595; 1.374–1.851), (1.513; 1.247–1.836) and (1.309; 1.028–1.665) respectively. Nausea, isolation and back head pain were caused by migraine and these were found positively significant with the odds ratios and 95% confidence interval of odds ratios(1.290; 1.122–1.484), (1.882; 1.617–2.190) and (1.285; 1.123–1.471) respectively.

**Conclusions:**

Stress, physical Activities and Menstruation increase the risk of migraine but weight loss, Breakfast, lunch, thirst, injury and Second trimester during pregnancy reduce the risk of migraine.

## Background

Migraine is the most common and typical cripple issue of the brain with the neurological cause of disability in the world [1]. International Classification of Headache Disorders *(*ICHD-3) is highly useful (96 and 98% respectively) than ICHD-3 beta for the diagnosis of migraine with aura and with typical aura [2]. The odds of touring headache emergency departments are significantly lower in Germany, France and UK as compared to Canada and Australia [3]. Migraine is a disabling disorder that occurs three times more in women than men. About 18% of women and 6% of men suffer from migraine in the United States [4]. Migraine is more common in women of middle age than men [5]. Women who experience Probable Migraine with Aura (PMWA) and smoke as well as using oral contraceptives methods have chances of having a stroke 7 times more than other women with PMWA [6]*.* Family history is the most important and consistent risk factor of migraine [7]. Migraine is nearly 3% of all the prevalent diseases of disability worldwide [8]. The composition of white matter varies in patients with chronic migraine (CM) and episodic migraine (EM) [9]. In episodic migraine (EM) and chronic migraine (CM), the expenditure of treatment is significantly higher for women than men. The annual direct expenditure of treatment for chronic migraine is 4.8 fold higher than that of episodic migraine [10].

Education, environmental display, travel, and oral contraceptives exposure nearly 75% of the underlying risks [11]. Migraine is a risk factor for ischemic stroke in young women and ischaemic stroke is strongly associated with migraine, both migraine without aura and migraine with aura [12]*.* In migraineurs, women, contemporary use of oral contraceptives, high blood pressure or smoking are a great effect on the odd*s* ratios for ischemic stroke associated with migraine in young women [13]. Migraineurs with aura can suffer from at least one attack per week and are likely to low intake of chocolate, ice cream and processed meats [14]. Weight loss may help to alleviate migraine in obese individuals. The relation of weight loss to those changes was assessed and the patients reporting moderate to severe disability decreased from 12(50.0%) before bariatric surgery to 3 (12.5%) after bariatric surgery [15]. Women with menstrual migraine experience higher headache intensity during early pregnancy and postpartum as compared with those without menstrual migraine [16]. Perceived stress affects greatly the quality of life. The relationship between stress and migraine is quite strong. Mean Perceived stress scale score is higher in chronic migraine patients than those in controls, after adjusting for education and anxiety [17]. Migraine is more common and related among depressed people, especially in females and in married women [18]. The prevalence of the most prevalent primary headaches, including migraine and tension-type headache, peaks between the ages of 20 and 40 and then decreases as people become older [19]. The survey of Korean patients with migraine illustrates that there are significant problems and unsatisfied needs to diagnosis, awareness, and treatment. The significant levels of disability, pain severity, and reduced quality of life are accomplished by Korean patients with migraine due to the burden of disease [20]. During the COVID-19 pandemic, the impact and severity of migraine are increased due to many risk factors such as disruption of sleep, anxiety and depression, dietary habits, lack of communication with treating neurologists and working [21]. During COVID-19 lockdown migraine symptoms are improved in the Netherlands due to lifestyle changes that can significantly improve the course of migraine [22]. The pain of migraine is higher among those individuals that can be influenced by complex emotional perception. Pain-related anxiety plays an important role in headache-related disability [23]. More details can be seen in [24–34].

This study was conducted to identify different risk factors of migraine in females and to identify the relationship between risk factors and migraine. Finally, to create awareness among females about risk factors of migraine.

## Methods

### Study design

This case-control study was performed to look at the risk factors of migraine in females. The network sampling method was used to collect data about migraine patients from the household, working, married women’s and female students. Samples were drawn from universities, colleges, parks, urban and rural areas due to non-probability sampling. The cross-sectional study was performed at Lady Willingdon, Lady Aschen, Gosh-e-Shifa, and Mayo hospitals from September 2019 to March 2020. In this study 315 controls those who were not affected due to migraine and 740 cases those who were affected due to migraine. We were taken great effort to use strong statistical procedures and stringent selection criteria to minimize bias and make sure that our control group is as representative as possible of the larger population of females in Pakistan, even if the size of the control group is lower. In this case– control study 315 age and gender-matched controls were included.

### Inclusion criteria

The inclusion criteria are a standard that is used to determine whether cases or controls will be included in the research. Below is a list of this requirement.

In this study, patients of various ages were enrolled.

The question was are you suffering from migraine?

Responses on the Likert Scale.

1 = “No” (I don’t have a migraine).

2 = “Rarely” (I sometimes get minor migraine symptoms).

3 = “Sometimes” (I occasionally but sporadically suffer migraine symptoms).

4 = “Frequently” (I frequently suffer from migraine symptoms).

5 = “Always” (I get severe migraine symptoms all the time).

For cases: Respondents who indicate on the Likert scale that they “Frequently” (4) or “Always” (5) get migraine symptoms.

For controls: Respondents who choose “No” (1), “Rarely” (2), or “Sometimes” (3) on the Likert scale serve as controls [35].

**Fig. 1:**
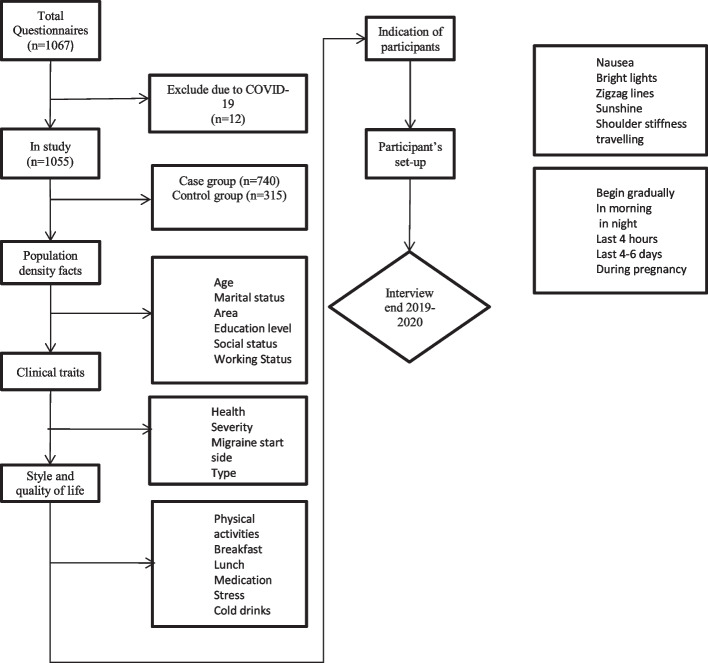
Flow chart depicting the inclusion of participants in the study

Flow chart depicting the inclusion of participants in the study is shown in Fig. 1.

### Instrument of the study

To gather information, a self-designed questionnaire was developed for both cases and controls [36]. The reliability of the questionnaire, as determined by Cronbach’s Alpha, was 0.95. A questionnaire is a set of written questions that are utilized with the end goal of information collection or statistical study. The questions included in the questionnaire, cover all the conceivable risk factors which include demographic variables: age, marital status, area, education level, social status, working status; clinical variables: health, severity, migraine start side, more than one type, physical activities, medication, last 4 hours, last 4–6 hours, last between 4and 72 hours; and symptoms variables: stress, upset, nausea, bright light, sunshine, yawing, zigzag lines, breakfast, lunch, travelling, shoulder stiffness in summer, in winter, quiet room cold drinks, menstrual cycle. The direct and face to face personal interviewing method was adopted from the cases and controls in hospitals, urban as well as rural areas [36]. 28 independent variables were included in the study, and the dependent variable is the suffering from migraine.

### Data collection

The network sampling method was used for data collection. The work of data collection started after the research issue was characterized and the research design was worked out. Data was gathered via questionnaire and face to face interview method. In this study 182 patients were under the age of 18 years whose data were collected through verbal consent from their parents, siblings and doctors. The total sample size was 1067 with a 95% confidence interval and 0.03 margin of error but the data collection procedure was stopped due to COVID-19 lockdown and collect total sample size was 1055 subjects. It is indicated by n. A very small number of samples were collect nearly 4 to 5 samples during lockdown with stringent precautionary measures.

### Significance of the study

The researcher truly expects that this review will be useful and can give a contribution to the certain gathering, as follow,

i. It will be valuable for a new researcher to sort out new factors influencing this disease.
ii. It might be advantageous for an overall population that they can take on these safety measures for keeping away from this life harming disease.
iii. It will be useful for strategy creators that they can carry out such strategies which will be life-saving and prevent migraine.
iv. It will be helpful for clinical staff that they can find out its starting point and guide their patients in a better way.
v. It will helpful for patients to prevent risk factors of migraine.

### Data analysis

After the data collection, the next and the most troublesome task was to analyze the information and sort out the outcomes. The software SPSS was utilized for the information processing and analysis. Descriptive and analytical outcomes were calculated, utilizing different statistical techniques, which include percentages, frequencies, Chi-square and binary logistic regression model. To determine the *p*-value and to look for a relationship between migraine and risk factors, the Chi-Square test was used [37]. Since the variables in the QOL section are categorical and chi-square handles qualitative variables, we were utilized it there. The distribution difference of a few chosen variables between migraine patients and healthy controls was assessed using the chi-square test. In regression analysis included Odds Ratio, 95% Confidence Interval, and *p*-values. The later results were considered significant when *p* < 0.05.

## Results

In this study of 1055 subjects, out of which 740 were cases and 315 were controls. Only females were considered. The analysis was conducted in which descriptive and analytical part was performed for explaining the different risk factors of the disease.

### Population density facts

The below table shows that among 740 patients with migraine, the counts (percentages) of 21–30 age interval was 316(42.70%), 386(52.16%) were married and 286(38.65%) have a graduate level. In urban areas, the counts (percentages) of cases and controls are 529(71.49%) and 206(65.40%), respectively. Rupees from Pakistan were used as payment. Income was used to determine social class, which was then separated into three groups: lower class (income < 20,000), middle class (Income 20,000–10,000), and high class (Income 10,000–100,000) [36]. In the case of patients with migraine, the counts (percentages) in the upper, middle and lower class are 30(4.05%), 690(93.24%) and 20(2.70%), respectively. Most of the cases belong to middle class. In cases most of the participants i.e., 529(71.49%) belongs to urban areas. According to a recent update on acute and preventative therapies for migraine in children and adolescents, only around 5% of American children and adolescents who are 10 years old suffer from migraines [7]. In order to provide total openness in our data presentation, we have included an age category for individuals who are younger than 10 years old. Complete population density facts are given below in Table 1.

**Table 1:** Population Density Facts of the Participants

Variables	Categories	Migraine				
		Yes	No	IRR	Chi-square	*p*-value
Age	< 10	1 (0.13)	0 (0)			0.000
	11–20	182(24.60)	100(31.75)	1.82		
	21–30	316(42.70)	123(39.05)	2.569106		
	31–40	164 (22.16)	59 (18.73)	2.779661		
	41–50	62 (8.34)	25 (7.93)	2.48		
	> 50	15 (2.03)	8 (2.54)	1.875		
Marital Status	Married	386 (52.16)	134 (42.54)	2.880597	8.553	0.036
	Single	340 (45.94)	173(54.92)	1.965318		
	Divorced	5(0.67)	2(0.63)	2.5		
	Widowed	9(1.22)	6(1.90)	1.5		
Area	Rural	211(28.51)	109(36.60)	1.93578	3.877	0.05
	Urban	529(71.49)	206(65.40)	2.567961		
Education Level	Metric	96(12.97)	34(10.79)	2.823529	8.706	0.121
	Intermediate	150(20.27)	55(17.46)	2.727273		
	Graduation	286(38.65)	133(42.22)	2.150376		
	Post graduation	156(21.08)	75(23.81)	2.08		
	Uneducated	52(7.03)	18(5.71)	3.4		
Social Status	**Upper class**	**30(4.05)**	**6(1.90)**	**0.333333**	**4.401**	**0.111**
	Middle class	690(93.24)	304(96.51)	5		
	Lower class	20(2.70)	5(1.59)	2.269737		
Working Status	Yes	316(42.70)	110(34.92)	4	5.558	0.018
	No	424(57.29)	205(65.08)	2.872727		

### Clinical traits of participants

Among 740 cases, most of the participants had an average of 394(53.24%) health in general, this means that mostly females with migraine are in general health, neither in good health nor in bad health. 334(45.13%) has moderate severity of migraine and 306(41.35%) have right side migraine start. The participants 180(24.32%) have more than one type of migraine and 550(74.32%) prefer a quiet room during migraine. Table 2 shows statistically significant associations of migraine with health, severity, migraine start, types and quiet room.

**Table 2:** Clinical Traits of Participants

Variables	Categories	Migraine				
		Yes	No	IRR	Chi-square	*p*-value
Health in general	Excellent	29(3.92)	31(9.84)	0.935484	22.363	0.000
	Good	282(38.11)	138(43.81)	2.043478		
	Average	394(53.24)	129(40.95)	3.054264		
	Poor	35 (4.73)	17(5.39)	2.058824		
	None	17(2.29)	284(90.16)	11.66667		
Severity of migraine	Mild	105(14.19)	9(2.86)	41.75	801.09	0.000
	Moderate	334(45.13)	8(2.54)	21.07692		
	Severe	274(37.02)	13(4.13)	0.094737		
	Others	27(3.65)	285(90.48)	42.8		
From where your migraine start	Left side	214(28.92)	5(1.59)	27.81818	867.57	0.000
	Right side	306(41.35)	11(3.49)	15.92308		
	Both sides	207(27.97)	13(4.13)	0.041958		
	Others	12(1.62)	286(90.79)	53.66667		
More than one type of migraine	Yes	161(21.75)	3(0.95)	20	319.25	0.000
	Sometimes	180(24.32)	9(2.86)	39		
	Often	117(15.81)	3(0.95)	7.5		
	Rarely	30(4.05)	4(1.27)	0.851351		
	No	252(34.05)	296(93.97)	8.461538	467.57	0.000
Prefer a quiet room during migraine	Yes	550(74.32)	65(20.63)	4.222222		
	Sometimes	76(10.27)	18(5.71)	4.333333		
	Often	39(5.27)	9(2.86)	2.923077		
	Rarely	38(5.135)	13(4.13)	0.17619		
	No	37(5.00)	210(66.67)	0.935484		

### Style and quality of life

Among 740 cases and 315 controls, the 5-points Likert scale was used for quality of life assessment in people. Counts (percentages) of physical activities are 357(48.24%), 511(69.05%) patients take breakfast every morning and 469(63.38%) take lunch every day. Maximum patients 468(63.24%) feel upset before migraine and 493(66.62%) patients respond that migraine start due to stress. The majority of the patients 302(40.81%) use the medicine. Table 3 shows that statistically significant associations of migraine with physical activities, stress, upset, medication, avoiding food, lunch, and cold drinks.

**Table 3:** Style and quality of life

Variables	Categories	Migraine				
		Yes	No	IRR	Chi-square	*p*-value
**Limit any physical activities**	Yes	357(48.24)	18(5.71)	19.83333	593.26	0.000
	Sometimes	189(25.54)	6(1.90)			
	Often	94(12.70)	13(4.12)	7.230769		
	Rarely	25(3.38)	4(1.27)	6.25		
	No	75(10.13)	274(86.98)	0.273723		
**Breakfast each morning**	Yes	511(69.05)	239(75.87)	2.138075	6.363	0.174
	Sometimes	71(9.59)	28(8.89)	2.535714		
	Often	48(6.49)	14(4.44)	3.428571		
	Rarely	23(3.10)	5(1.59)	4.6		
	No	87(11.75)	29(9.21)	3		
**Lunch each day**	Yes	469(63.38)	235(74.60)	1.995745	30.134	0.000
	Sometimes	132(17.84)	29(9.20)	4.551724		
	Often	37(5.00)	11(3.49)	3.363636		
	Rarely	41(5.54)	3(0.95)	13.66667		
	No	144(19.46)	290(92.06)	1.648649		
**Are you on medication**	Yes	302(40.81)	10(3.17)	30.2	242.75	0.000
	Sometimes	96(12.97)	8(2.54)	12		
	Often	49(6.62)	17(5.39)	2.882353		
	Rarely	38(5.13)	14(4.44)	2.714286		
	No	255(34.46)	266(84.44)	0.958647		
**Feel upset before migraine**	Yes	468(63.24)	18(5.71)	26	555.09	0.000
	Sometimes	104(14.05)	17(5.39)	6.117647		
	Often	67(9.05)	6(1.90)	11.16667		
	Rarely	22(2.97)	9(2.86)	2.444444		
	No	79(10.67)	265(84.12)	0.298113		
**Migraine start due to stress**	Yes	493(66.62)	16(5.08)	30.8125	614.19	0.000
	Sometimes	108(14.59)	12(3.81)	9		
	Often	61(8.24)	12(3.81)	5.083333		
	Rarely	20(2.70)	15(4.76)	1.333333		
	No	58(7.83)	260(82.53)	0.223077		
**Do you know the type of food to avoid**	Yes	342(46.21)	15(4.76)	22.8	266.45	0.000
	Sometimes	92(12.43)	21(6.66)	4.380952		
	Often	40(5.40)	9(2.86)	4.444444		
	Rarely	36(4.86)	3(0.95)	12		
	No	230(31.08)	267(84.76)	0.861423		
**Cold drinks cause migraine**	Yes	79(10.67)	14(4.44)	5.642857	75.84	0.000
	Sometimes	61(8.24)	4(1.27)	15.25		
	Often	51(6.89)	8(2.54)	6.375		
	Rarely	66(8.91)	3(0.95)	22		
	No	483(65.27)	286(90.79)	1.688811		

### Indication of participants

Among 740 patients with migraine 330(44.59%) reported that they usually suffer from nausea/vomiting, 419(56.62%) patients effect by bright light, and 500(67.57%) are affected by noise. 206(27.84%) patients said that they see zigzag lines before migraine, 338(45.67%) have a sunshine cause. Most of the patients 362(48.92%) have shoulder stiffness. Table 4 shows that a significant association of nausea, bright light, noise, lines, sunshine, shoulder stiffness, yawing and travelling with migraine.

**Table 4:** Indication of Participants

Variables	Categories	Migraine				
		Yes	No	IRR	Chi-square	*p*-value
Suffer from nausea?	Yes	330(44.59)	20(6.34)	4.59375	251.51	0.000
	Sometimes	147(19.86)	32(10.16)	5		
	Often	50(6.76)	10(3.17)	1.347826		
	Rarely	31(4.19)	23(7.30)	0.791304		
	No	182(24.59)	230(73.01)	9.97619		
Does the bright light affect you?	Yes	419(56.62)	42(13.33)	2.542373	261.76	0.000
	Sometimes	150(20.26)	59(18.73)	2.037037		
	Often	55(7.43)	27(8.57)	1.608696		
	Rarely	37(5)	23(7.30)	0.481707		
	No	79(10.67)	164(52.06)	8.928571		
Do noises affect you?	Yes	500(67.57)	56(17.78)	2.416667	312.66	0.000
	Sometimes	116(15.67)	48(15.24)	1.155556		
	Often	52(7.03)	45(14.28)	1.2		
	Rarely	24(3.24)	20(6.35)	0.328767		
	No	48(6.45)	146(46.34)	8.956522		
See zigzag lines before migraine?	Yes	206(27.84)	23(7.30)	10.66667	179.72	0.000
	Sometimes	160(21.62)	15(4.76)	3.823529		
	Often	65(8.78)	17(5.39)	3.764706		
	Rarely	64(8.65)	17(5.39)	1.00823		
	No	245(33.11)	243(77.14)	11.65517		
Sunshine trigger?	Yes	338(45.67)	29(9.20)	5.913043	263.21	0.000
	Sometimes	136(18.38)	23(7.30)	3.666667		
	Often	55(7.43)	15(4.76)	2.619048		
	Rarely	55(7.43)	21(6.67)	2.308176		
	No	367(49.59)	159(50.48)	6.581818		
Shoulder stiffness/neck pain?	Yes	362(48.92)	55(17.46)	5.275862	250.65	0.000
	Sometimes	153(20.67)	29(9.20)	3.73913		
	Often	86(11.62)	23(7.30)	2		
	Rarely	40(5.40)	20(6.34)	0.526596		
	No	99(13.37)	188(59.68)	6.466667		
Yawing before migraine?	Yes	194(26.21)	30(9.52)	29.6	199.01	0.000
	Sometimes	148(20)	5(1.58)	8.777778		
	Often	79(10.67)	9(2.85)	5.2		
	Rarely	52(7.02)	10(3.17)	1.022989		
	No	267(36.08)	261(82.85)	8.044444		
Migraine during travelling?	Yes	362(48.91)	45(14.28)	3.179487	212.79	0.000
	Sometimes	124(16.75)	39(12.38)	3.047619		
	Often	64(8.64)	21(6.67)	3.315789		
	Rarely	63(8.51)	19(6.03)	0.664921		
	No	127(17.16)	191(60.63)	16.5		

### Participants set-up

Among 740 cases, the majority of patients 339(45.81%) respond to migraine beginning gradually, 237(22.5%) respond to migraine beginning in the morning and 239(22.7%) respond to migraine beginning in the night sometimes. 272(36.76%) patients respond that they have migraine last between 4 and 72 hours. During summer 354(47.84%) are affected and during winter 289(39.05%) patients are affected. A significant association begins gradually, in the morning, in the night, last 4 hours, last 4–6 days, last 4–72 hours, during summer, during winter, during the menstrual cycle, and pregnancy with migraine. The participants set-up is shown in Table 5.

**Table 5:** Participants Set-Up

Variables	Categories	Migraine				
		Yes	No	IRR	Chi-square	*p*-value
Does your migraine typically begin gradually?	Yes	339(45.81)	13(4.13)	26.07692	597.15	0.000
	Sometimes	196(26.48)	8(2.54)	24.5		
	Often	73(9.86)	4(1.27)	18.25		
	Rarely	41(5.54)	3(0.95)	13.66667		
	No	91(12.29)	287(91.11)	0.317073		
Begin in the morning?	Yes	231(31.21)	6(1.90)	38.5	412.18	0.000
	Sometimes	195(26.35)	6(1.90)	32.5		
	Often	79(10.67)	2(0.63)	39.5		
	Rarely	48(6.45)	8(2.54)	6		
	No	187(25.27)	293(93.01)	0.638225	481.99	0.000
Begin in the night?	Yes	210(28.38)	4(1.27)	52.5		
	Sometimes	225(30.40)	14(4.44)	16.07143		
	Often	95(12.48)	3(0.95)	31.66667		
	Rarely	66(8.92)	4(1.27)	16.5		
	No	144(19.46)	290(92.06)	0.496552		
Duration of your migraine usually last 4 hours?	Yes	227(30.67)	8(2.54)	28.375	397.19	0.000
	Sometimes	196(26.49)	6(1.90)	32.66667		
	Often	104(14.05)	10(3.17)	10.4		
	Rarely	37(5)	8(2.54)	4.625		
	No	176(23.78)	283(89.84)	0.621908		
Does migraine duration usually last 4–6 days?	Yes	220(29.72)	5(1.59)	44	350.31	0.000
	Sometimes	151(20.40)	6(1.90)	25.16667		
	Often	92(12.43)	8(2.54)	11.5		
	Rarely	58(7.83)	5(1.59)	11.6		
	No	219(29.59)	315(100)	0.695238		
Does migraine last between 4 and 72 hours?	Yes	272(36.76)	12(3.81)	22.66667	301.27	0.000
	Sometimes	105(14.19)	6(1.90)	17.5		
	Often	59(7.97)	3(0.95)	19.66667		
	Rarely	59(7.97)	6(1.90)	9.833333		
	No	245(33.10)	288(91.43)	0.850694		
Migraine during summer?	Yes	354(47.84)	14(4.44)	25.28571	420.84	0.000
	Sometimes	130(17.56)	19(6.03)	6.842105		
	Often	90(12.16)	8(2.53)	11.25		
	Rarely	45(6.08)	19(6.03)	2.368421		
	No	121(16.35)	255(80.95)	0.47451		
Migraine during winter?	Yes	289(39.05)	12(3.80)	24.08333	302.08	0.000
	Sometimes	139(18.78)	20(6.34)	6.95		
	Often	71(9.59)	13(4.12)	5.461538		
	Rarely	59(7.97)	14(4.44)	4.214286		
	No	182(24.59)	256(81.26)	0.710938		
Migraine during Menstrual Cycle?	Yes	281(37.97)	9(2.85)	31.22222	331.40	0.000
	Sometimes	163(22.02)	11(3.49)	14.81818		
	Often	52(7.02)	11(3.49)	4.727273		
	Rarely	40(5.40)	7(2.22)	5.714286		
	No	204(27.56)	277(87.93)	0.736462		
Affected 2 days before bleeding start until 3 days?	Yes	251(33.92)	8(2.53)	31.375	347.65	0.000
	Sometimes	176(23.78)	10(3.17)	17.6		
	Often	62(8.38)	6(1.90)	10.33333		
	Rarely	45(6.08)	7(2.22)	6.428571		
	No	206(27.83)	284(90.15)	0.725352		
Migraine during pregnancy?	Yes	159(21.48)	13(4.12)	12.23077	91.36	0.000
	Sometimes	50(6.75)	3(0.95)	16.66667		
	Often	23(3.10)	1(0.31)	23		
	Rarely	6(0.81)	1(0.31)	6		
	No	156(21.08)	118(37.46)	1.322034		
	Not applicable	346(46.75)	179(56.82)	1.932961		

A binary logistic regression model was run and under this model, regression coefficients, odds ratio, *p*-value, and 95% confidence interval for odds ratio are computed. At the 5% level of significance, the *p*-value is compared. Binary logistic regression described that the most significant risk factors of migraine were weight loss, activities, morning, injury, stress, nausea, Breakfast, lunch, summer, quiet room, pain, thirst, cold drinks, food and second trimester. The statistics are shown in Table 6.

**Table 6:** Model Coefficient, Odds Ratios and 95% CI’s for Odds Ratios

Variables	β	S.E	Wald	df	Sig.	Exp(β)	95% CI for Exp(β)	
							Lower	Upper
Weight loss	−0.575	0.107	29.102	1	0.000	0.563	0.457	0.694
Begin gradually	0.195	0.108	3.282	1	0.070	1.215	0.984	1.500
Activities	0.336	0.113	8.859	1	0.003	1.399	1.122	1.746
Morning	0.269	0.123	4.784	1	0.029	1.309	1.028	1.665
Upset	0.118	0.119	0.980	1	0.322	1.125	0.891	1.420
Injury	−0.376	0.105	12.830	1	0.000	0.687	0.559	0.844
Stress	0.412	0.123	11.241	1	0.001	1.510	1.187	1.922
Last 4–72 hours	−0.263	0.126	4.393	1	0.036	0.768	0.601	0.983
Nausea	0.255	0.071	12.720	1	0.000	1.290	1.122	1.484
Travelling	−0.134	0.071	3.531	1	0.060	0.875	0.761	1.006
Breakfast	−0.206	0.085	5.912	1	0.015	0.814	0.689	0.961
Lunch	−0.532	0.087	37.072	1	0.000	0.588	0.495	0.697
Summer	0.467	0.076	37.854	1	0.000	1.595	1.374	1.851
Winter	0.087	0.075	1.350	1	0.245	1.091	0.942	1.264
Quiet Room	0.632	0.077	66.720	1	0.000	1.882	1.617	2.190
Pain	0.251	0.069	13.262	1	0.000	1.285	1.123	1.471
Thirst	−0.185	0.075	6.037	1	0.014	0.832	0.718	0.963
Perfume	−0.016	0.073	0.050	1	0.823	0.984	0.852	1.135
Cold Drinks	−0.476	0.088	29.159	1	0.000	0.622	0.523	0.739
Food like cheese	−0.249	0.088	8.074	1	0.004	0.780	0.657	0.926
Periods	0.184	0.097	3.578	1	0.059	1.202	0.993	1.455
Menstrual cycle	0.414	0.099	17.597	1	0.000	1.513	1.247	1.836
Pregnancy	0.162	0.096	2.868	1	0.090	1.176	0.975	1.418
Second	−0.351	0.118	8.838	1	0.003	0.704	0.559	0.887

Logit (p) = −0.575*weight Loss + 0.336*Activities + 0.269*Morning – 0.376*Injury + 0.412*Stress + 0.255*Nausea – 0.206*Breakfast - 0.532*Lunch + 0.467*summer + 0.632*Quiet + 0.251*Pain – 0.185*Thirst - 0.476*Cold drinks – 0.249*Food + 0.414* Menstrual cycle – 0.351*Second.

## Discussions

It was observed from the data that nausea is found to be positively significant with the odds ratios and 95% confidence interval of odds ratios (1.290; 1.122–1.484). Out of 740 patients, 330(44.59%) patients respond that they usually suffer from nausea during migraine. In migraine, nausea is a centrally driven symptom that occurs as a premonitory symptom independent of pain and is associated with activation of brain structures known to be involved in nausea [38]. It was observed from the data that stress is found to be positively significant with the odds ratios and 95% confidence interval of odds ratios (1.510; 1.187–1.922). Out of 740 patients, 493(66.62%) patients respond that migraine start due to stress. Stress and migraine are reciprocally related. Migraine is more common and related among depressed people, especially females. The association between migraine and sleep disorders is complex and significant. Improvement of sleep is considered to reduce headache frequency and severity and reduce stress [10-7-30]. Whereas the menstrual cycle is found to be positively significant with the odds ratios and 95% confidence interval of odds ratios (1.513; 1.247–1.836). Women with menstrual migraine have higher headache intensity during early pregnancy and postpartum compared to those without menstrual migraine [16]. Activities, morning, nausea, summer, quiet and pain are found to be positively significant with the odds ratios and 95% confidence interval of odds ratios (1.399; 1.122–1.746), (1.309; 1.028–1.665), (1.290; 1.122–1.484), (1.595; 1.374–1.851), (1.882; 1.617–2.190) and (1.285; 1.123–1.471) respectively. While six factors in which injury, breakfast, lunch, thirst, drinks and second trimester are found to negatively significant with the odds ratios and 95% confidence interval of odds ratios (0.687; 0.559–0.844), (0.814; 0.689–0.961), (0.588; 0.495–0.697), and (0.704; 0.559–0.887), respectively. Weight loss is found to be negatively significant with the odds ratios and 95% confidence interval of odds ratios (0.563; 0.457–0.694). The relation of weight loss to those changes was assessed and the patients reporting moderate to severe disability decreased from 12(50.0%) before surgery to 3(12.5%) after surgery [15]. Second trimester during pregnancy is observed to be negatively significant with an odds ratio is 0.704 times higher. Around 60–70% of migraineurs experience improvement in migraine during pregnancy; in around 20% attacks disappear. If migraine was not improved by the end of the first trimester it is likely to continue throughout pregnancy and postpartum [16]. Another study described that a significant decrease in the duration of migraine (*p* < 0.001) during pregnancy as compared to before [7].

## Limitations of the study

The important limitation of the study was the sample size collected. The small sample size made it ambitious to conduct analysis. Many further limitations obtain in the interview and data collection previous to analysis. In hospitals and universities, some females refuse to allow for conducting the interview. In hospitals and rural areas, the language issue was confronted where a large number of females did not know the national language. Most of the females do not know about migraine but symptoms were matched so the analysis was difficult if data is unreliable. In our study, the final major limitation was the potential for subject to bias. As with any case-control study, it is conceivable that either cases or controls were differentially bound to subjects than the other.

## Conclusions

Stress, Activities, and menstrual cycle increase the risk of migraine but weight loss, Breakfast, and the Second trimester during pregnancy reduce the risk of migraine. The stress had 1.510 times higher of migraine Nausea with an odds ratio of 1.290 and 95% CI of odds ratio (1.122–1.484). So we conclude that the binary response variable suffering from migraine patients who are usually suffering from nausea is 1.290 times more chance than others. Pain (back of the head) with odds ratio 1.285 and 95% CI of odds ratio (1.123–1.471). So we conclude that the binary response variable suffering from migraine patients who are feeling pain on the back of the head is a 1.285 times more chance of getting the disease than others.

## Data Availability

The datasets generated and/or analyzed during the current study are not publicly available due to limitations of ethical approval involving the patient data and anonymity but are available from the corresponding author on reasonable request.

## References

[1] Puledda F, Messina R, Goadsby PJ (2017). An update on migraine: current understanding and future directions. J Neurol..

[2] Gobel CH, Karstedt SC, Munte TF, Gobel H, Wolfrum S, Lebedeva ER, Royl G (2020). ICHD-3 is significantly more specific than ICHD-3 beta for the diagnosis of migraine with aura and with typical aura. J Headache Pain..

[3] Sanderson JC, Devine EB, Lipton RB, Bloudek LM, Varon SF, Blumenfeld AM, Sullivan SD (2013). Headache-related health resource utilisation in chronic and episodic migraine across six countries. J Neurol Neurosurg Psychiatry..

[4] Peterlin BL, Gupta S, Ward TN, Macgregor A (2011). Sex matters: evaluating sex and gender in migraine and headache research. Headache..

[5] Scher AI, Gudmundsson LS, Sigurdsson S, Ghambaryan A, Aspelund T, Eiriksdottir G, Launer LJ (2009). Migraine headache in middle age and late-life brain infarcts. JAMA..

[6] MacClellan LR, Giles W, Cole J, Wozniak M, Stern B, Mitchell BD, Kittner SJ (2007). Probable migraine with visual aura and risk of ischemic stroke: the stroke prevention in young women study. Stroke..

[7] Lateef TM, Cui L, Nakamura E, Dozier J, Merikangas K (2015). Accuracy of family history reports of migraine in a community-based family study of migraine. Headache..

[8] Leonardi M, Raggi A (2013). Burden of migraine: international perspectives. Neurol Sci..

[9] Planchuelo-Gomez A, Garcia-Azorin D, Guerrero AL, Aja-Fernandez S, Rodriguez M, de Luis-Garcia R (2020). White matter changes in chronic and episodic migraine: a diffusion tensor imaging study. J Headache Pain..

[10] Negro A, Sciattella P, Rossi D, Guglielmetti M, Martelletti P, Mennini FS (2019). Cost of chronic and journal of headache and pain.

[11] Ray BK, Paul N, Hazra A, Das S, Ghosal MK, Misra AK, Das SK (2017). Prevalence, burden, and risk factors of migraine: a community-based study from eastern India. Neurol India..

[12] Tzourio C, Tehindrazanarivelo A, Iglésias S, Alpérovitch A, Chedru F, Anglejan-Chatillon J, Bousser MG (1995). Case-control study of migraine and risk of ischaemic stroke in young women. BMJ..

[13] Chang CL, Donaghy M, Poulter N (1999). Migraine and stroke in young women: a case-control study. BMJ..

[14] Rist PM, Buring JE, Kurth T (2015). Dietary patterns according to headache and migraine status: a cross-sectional study. Cephalalgia..

[15] Bond D, Vithiananthan S, Nash J, Thomas J, Wing R (2011). Improvement of migraine headaches in severely obese patients after bariatric surgery. Neurology..

[16] Petrovski BE, Vetvik KG, Lundqvist C, Eberhard-Gran M (2018). Characteristics of menstrual versus non-menstrual migraine during pregnancy: a longitudinal population-based study. J Headache Pain..

[17] Milde-Busch A, Blaschek A, Heinen F, Borggrafe I, Koerte I, Straube A, von Kries R (2011). Associations between stress and migraine and tension-type headache: results from a school-based study in adolescents from grammar schools in Germany. Cephalalgia..

[18] Jat MI, Afridi MI, Amar W, Lal C (2018). Prevalence of migraine among patients of depressive disorder. Pak J Med Sci..

[19] Schramm S, Tenhagen I, Schmidt B (2021). Prevalence and risk factors of migraine and non-migraine headache in older people – results of the Heinz Nixdorf recall study. Cephalalgia..

[20] Kim BK, Chu MK, Yu SJ, Dell'Agnello G, Han JH, Cho SJ (2021). Burden of migraine and unmet needs from the patients' perspective: a survey across 11 specialized headache clinics in Korea. J Headache Pain..

[21] Al-Hashel JY, Ismail II (2020). Impact of coronavirus disease 2019 (COVID-19) pandemic on patients with migraine: a web-based survey study. J Headache Pain..

[22] Shimizu T, Sakai F, Miyake H, Sone T, Sato M, Tanabe S, Dodick DW (2021). Disability, quality of life, productivity impairment and employer costs of migraine in the workplace. J Headache Pain..

[23] Kim S, Bae DW, Park SG, Park JW (2021). The impact of pain-related emotions on migraine. Sci Rep..

[24] Haw NJ, Cabaluna IT, Kaw GE, Cortez JF, Chua MP, Guce K (2020). A cross-sectional study on the burden and impact of migraine on work productivity and quality of life in selected workplaces in the Philippines. J Headache Pain..

[25] Jedynak J, Eross E, Gendolla A, Rettiganti M, Stauffer VL (2021). Shift from high-frequency to low-frequency episodic migraine in patients treated with Galcanezumab: results from two global randomized clinical trials. J Headache Pain..

[26] Kajal M, Malik M, Kumari R (2017). Correlation of stress with migraine-a review. Int J Curr Res Rev..

[27] Li C, Li Y, Ma M, Zhang Y, Bao J, Ge W, He L (2021). The impact of COVID-19 pandemic on headache symptoms and drug withdrawal among patients with medication overuse headache: a cross-sectional study. J Headache Pain..

[28] MacGregor EA (2007). Migraine in pregnancy and lactation: a clinical review. J Fam PlannReprod Health Care..

[29] Steiner TJ, Stovner LJ, Jensen R, Uluduz D, Katsarava Z, The L, Burden: the Global Campaign against, H (2020). Migraine remains second among the world's causes of disability, and first among young women: findings from GBD2019. J Headache Pain..

[30] Tiseo C, Vacca A, Felbush A, Filimonova T, Gai A, Glazyrina T, European Headache Federation School of Advanced, S (2020). Migraine and sleep disorders: a systematic review. J Headache Pain..

[31] Verhagen IE, van Casteren DS, de Vries Lentsch S, Terwindt GM (2021). Effect of lockdown during COVID-19 on migraine: a longitudinal cohort study. Cephalalgia..

[32] Wilkes MJ, Mendis MD, Bisset L, Leung FT, Sexton CT, Hides JA (2021). The prevalence and burden of recurrent headache in Australian adolescents: findings from the longitudinal study of Australian children. J Headache Pain..

[33] Zahid M, Sthanadar A, Kaleem M, Latif M, Sthanadar I, Ali P, Sthanadar I, Ismail M, Imtiaz N, Shah M. Prevalence and perceptions about migraine among students and patients in Khyber Pakhtunkhwa Province. Pakistan; 2014.

[34] Krzych ŁJ, Lach M, Joniec M, Cisowski M, Bochenek A (2018). The Likert scale is a powerful tool for quality of life assessment among patients after minimally invasive coronary surgery. Kardiochirurgia i torakochirurgia polska = Polish J Cardio-Thorac Surg..

[35] Chu S, Wu Z, Wu Z, Wu J, Qian Y (2021). Association between insomnia and migraine risk: a case-control and bidirectional Mendelian randomization study. Pharmacogenomics Pers Med..

[36] Ahmad MR, Pervaiz MK (2010). Risk factors of urinary bladder cancer in Peshawar region of Khyber Pukhtoonkhawa. J Ayub Med Coll, Abbottabad : JAMC..

[37] Fatima T, Iftikhar S, Qureshi IH (2020). Association between hyperuricemia and ischemic stroke: a case-control study. J Coll Physicians Surg--Pak : JCPSP..

[38] Maniyar FH, Sprenger T, Schankin C, Goadsby PJ (2014). The origin of nausea in migraine-a PET study. J Headache Pain..

